# Optimization of fermentation conditions for enhanced L-arginase production by *Alcaligenes aquatilis* BC2 using response surface methodology

**DOI:** 10.1016/j.jgeb.2025.100591

**Published:** 2025-10-15

**Authors:** Birhan Getie Assega, Kefyalew Ayalew Getahun, Tamene Milkessa Jiru, Tsehayneh Geremew Yohannes, Mulugeta Aemero, Berhanu Andualem

**Affiliations:** a Department of Environmental and Industrial Biotechnology, Institute of Biotechnology, University of Gondar, Gondar, Ethiopia; b Department of Pharmacology, School of Pharmacy, College of Medicine and Health Sciences, University of Gondar, Gondar, Ethiopia; c Department of Medical Parasitology, SBLS, College of Medicine and Health Sciences, University of Gondar, Gondar, Ethiopia

**Keywords:** *Alcaligenes aquatilis* BC2, L-arginase, Optimization, Response surface methodology, Anticancer

## Abstract

L-arginase-based enzyme therapy, which depletes L-arginine by converting it to L-ornithine and urea, selectively inhibits the growth of L-arginine-dependent cancer cells with low toxicity. This approach shows promise as a novel cancer treatment. This research used Response Surface Methodology (RSM) to enhance L-arginase production by *Alcaligenes aquatilis* BC2, which was isolated from an Ethiopian soda lake. The Plackett-Burman Design was used to screen eight factors that influence L-arginase production and identified arginine concentration, peptone concentration, and incubation temperature as the most significant variables. The central composite design analysis demonstrated that the optimized conditions of 1.75 % L-arginine concentration, 3 % peptone concentration, and an incubation temperature of 37.5 °C enhance L-arginase production from a baseline of 92.45 U/mL to an optimized yield of 288.79 U/mL. This represents a 3.1-fold increase under the optimized conditions.

The model was developed based on 20 experimental runs, demonstrating excellent fit with R2 = 0.9974 and a significant F-value of 420.28 (p < 0.0001). Additionally, the lack-of-fit test was conducted and found to be non-significant (F-value = 4.18, p = 0.0714), further supporting the model’s predictive strength. This investigation showed that applying statistical design to optimize fermentation conditions leads to increased production of L-arginase, thereby advancing enzyme-based therapeutic practices and highlighting statistical optimization as essential for bioprocess development.

## 1 Introduction

Cancer is the leading cause of mortality worldwide; therefore, the exploration of novel therapeutic strategies is essential to combat this devastating disease.^1^ Over the decades, our understanding of cancer biology has greatly changed, enabling the development of many treatment strategies including surgical intervention, radiation therapy, and chemotherapy. However, since regular treatments often have several limitations and side effects, innovative solutions with greater efficacy and fewer adverse effects are urgently needed.^2^ Together with the specific metabolic weaknesses of many cancer cells, especially those displaying arginine auxotrophy, the use of low molecular weight enzymes with particular activities and minimal toxicity has given enzyme based treatments as a promising alternative in cancer treatment.^3^, ^4^

Enzyme-based anticancer therapies have gained significant attention due to their ability to selectively target metabolic dependencies of cancer cells. Several therapeutic enzymes, including L-methioninase, L-asparaginase, and glutaminase, have been explored for their capacity to deplete amino acids critical for tumor survival^5^. L-methioninase targets methionine, an essential amino acid for many tumors; L-asparaginase depletes asparagine, particularly effective in treating acute lymphoblastic leukemia; and glutaminase disrupts glutamine metabolism, which is vital for cancer cell proliferation.^6^ Among these, L-arginase has emerged as a promising candidate due to its ability to hydrolyze arginine, a semi-essential amino acid required by various arginine-auxotrophic tumors. Compared to other enzymes, L-arginase offers distinct advantages in terms of substrate specificity and potential therapeutic window, which motivated its selection for optimization in this study.

L-arginase offers a compelling therapeutic approach by catalyzing the hydrolysis of L-arginine into L-ornithine and urea.^7^ Cancer cells that lack L-argininosuccinate synthetase 1 (ASS1), depend on an external source of L-arginine for growth because they are unable to synthesize it, unlike healthy cells.^8^ The depletion of L-arginine by this enzyme starves cancer cells, thereby slowing their growth and proliferation. Husain et al.^9^ reported the therapeutic effectiveness of L-arginase in several types of cancer, including prostate cancer, leukemia, glioblastoma, breast cancer, and non-Hodgkin’s lymphoma. Riess et al.^10^ also demonstrated the impact of L-arginase on hepatocellular carcinoma and malignant melanoma whereas Yang et al.^11^ indicate their effect on pancreatic cancer.

Beyond its anticancer effects, L-arginase is involved in the production of L-ornithine, a significant intermediary in the urea cycle that facilitates the detoxification of excess ammonia. L-ornithine has been demonstrated as useful in the treatment of liver disorders and in safeguarding the heart from endotoxin-induced shock, thereby underscoring the therapeutic adaptability of L-arginase.^12^

While L-arginase is found in plants and animals, microbes offer a scalable, cost-effective production platform with easy cultivation, shorter growth times, and adaptability for genetic optimization. The ability of marine microbes to adapt to harsh conditions gives them a distinct edge and leads to the production of powerful and innovative proteins.^13^ Since the saline character of the marine environment is chemically comparable to that of human blood plasma, enzymes sourced from marine environments may be more effective and have fewer adverse effects.^14^

According to Mahjoubi et al.,^15^ *Alcaligenes aquatilis* is a marine bacterium that has garnered attention for its ability to break down hydrocarbons and produce biosurfactants. Additionally, recent studies have reported that *A. aquatilis* strains can produce L-methioninase, an enzyme with notable anticancer potential, demonstrating enzyme activity even under stress conditions such as high salinity and the presence of heavy metals.^16^ This study is the first to report application of statistical experimental designs, specifically Response Surface Methodology (RSM), enables more efficient and precise optimization of fermentation conditions. This approach significantly enhances L-arginase yield from *A. aquat*ilis BC2, thereby contributing novel advancements in enzyme production technology relevant for anticancer therapeutic applications. The optimization of fermentation conditions is crucial for the successful industrial application of microbial enzymes.^17^

Classical optimization techniques that focus on a single factor at a time are frequently laborious and miss interacting effects across variables.^18^ Although it has a direct impact on product concentration, yield, and downstream processing, developing a suitable production medium and conditions is crucial for enhancing the effectiveness and productivity of bioactive microbial metabolite fermentation processes.

Statistical experimental designs, like Response Surface Methodology (RSM), offer a potent and effective way to improve culture conditions. RSM allows for the effective optimization of several variables with a small number of tests, saving time and money, by utilizing experimental designs such as central composite design.^19^

Optimization of L-arginase production is essential for enhancing production and lowering manufacturing costs, making it more accessible for therapeutic use. RSM is a set of statistical and mathematical methods used to design experiments, build models, evaluate the effects of factors, and determine optimal conditions for desired responses.^20^ It allows for the analysis of individual and combined effects of multiple variables, leading to the development of a mathematical model that accurately describes the process. This study aims to statistically optimize the production of anticancer L-arginase by Alcaligenes aquatilis BC2 using RSM.

## 2 Material and methods

### 2.1 Chemicals

All chemicals used for this study were obtained from Merc (Merc KGaA, Germany) and Hi Media Laboratories (India).

### 2.2 Microorganism and culture condition

#### 2.2.1 Strain isolation and screening

The bacterial strain used in this study was isolated from sediment and water samples collected from Lake Chitu, an Ethiopian soda lake. The isolation of bacteria was performed by using mineral arginine agar media (MAA) composition containing: 5 g/L KCl, 5 g/L MgSO_4_, 10 g/L KH_2_PO_4_, 1 g/L FeSO_4_, 1 g/L ZnSO_4_ and 10 g/L L-arginine, along with 20 g/L agar. The culture was incubated at 37 °C and pH 7 for 48 h.^21^ Primary screening for L-arginase activity was based on a qualitative colorimetric change of the medium, utilizing phenol red as a pH indicator, where bacteria producing L-arginase hydrolyze L-arginine to ornithine and urea, increasing medium pH and changing its color from yellow to pink.^22^ Colonies causing such color change were identified as potential L-arginase producers. Following isolation and initial screening, quantitative assays measuring urea production from L-arginine hydrolysis were conducted to confirm and select the highest L-arginase-producing isolates.

The culture slants were prepared using the aforementioned media, sub cultured at 14-day intervals, and kept at 4 °C for further optimization process.

#### 2.2.2 Molecular identification of bacterial isolate

Genomic DNA was extracted from the bacterial isolate using the Qiagen DNA extraction kit, and its quality was verified via NanoDrop spectrophotometry and 2 % agarose gel electrophoresis. The 16S rRNA gene was amplified using universal primers 27F and 1492R under standard PCR conditions.^23^ The resulting amplicons were purified and sequenced (Macrogen Europe BV, Netherlands). Sequence alignment was performed using BLAST. A phylogenetic tree was constructed using the neighbor-joining method in MEGA 13 with 1,000 bootstrap replicates.^24^

### 2.3 Production of L-arginase

L-arginase was produced by submerged fermentation. The chosen bacterial isolate, 1 % v/v (1 × 10^8^ cells/mL), was inoculated into a 250 mL Erlenmeyer flask containing a production medium of 10 g/L glucose, 1 g/L K_2_HPO_4_, and 5 g/L L-arginine, (pH of 7.0), and incubated at 37 °C with shaking at 200 rpm for 24 h.^25^ After being grown, the culture medium was spun at 10,000 rpm for half an hour at 4 °C. The resultant cell-free supernatant was used as crude enzyme. Beginning with the one factor at a time (OFAT) procedure, H, temperature, inoculum volume, substrate concentration, carbon and nitrogen sources, fermentation time, and agitation speed were optimized to achieve maximum enzyme production by the isolated bacterial strain (Table 3).

### 2.4 Estimation of L-arginase activity

The L-arginase activity assay was determined by measuring the amount of urea released in the reaction. When urea is heated in the presence of mixed color (diacetyl monoxime) and mixed acid reagent, it produces a pink-colored complex that was calculated colorimetrically using the Archibald method.^22^ In this method, 0.5 mL of L-arginase was activated for 10 min by adding 0.2 mL of glycine-NaOH and 0.1 mL of MnCl_2_ at 37 °C. 0.25 mM of L-arginine (0.1 mL) was added to the reaction mixture and incubated for 30 min at 37 °C. The reaction mixture was then stopped by the addition of 1 mL of 3 % perchloric acid. 1 mL of the reaction mixture was added to the mixed acid and diacetyl monoxime reagent. After boiling for 20 min, the absorbance of the sample and the blank were measured at 520 nm. A standard curve graph was prepared by measuring the absorbance of different concentrations (0.1, 0.2, 0.3, 0.4 and 0.5 mM) of standard urea. Enzyme without substrate and substrate without enzyme were used as blanks. 1 Unit (U) of L-arginase activity is defined as the amount of enzyme that catalyzes the production of 1 Âµmol of urea per minute under the assay conditions.

$$Enzyme\;activity\left(U/mL\right)=\frac{\mu moles\;of\;urea\;released}{Time\;of\;enzyme\;action\times volume\;of\;enzyme(mL)}$$

### 2.5 Optimization using statistical design

#### 2.5.1 Selection of variables for Plackett-Burman design (PBD)

The Plackett-Burman design is commonly employed for screening process variables to enhance enzyme production.^26^ It relies on a first-order model that assumes no interactions between factors. Based on a review of the literature, previous experimental data, and their known or suspected effects on microbial growth and metabolite production, eight variables were selected for the Plackett-Burman design. As the main sources of carbon and nitrogen, peptone and maltose (X_1_ and X_2_) were selected from the OFAT results of the present study. These substances are frequently shown to affect biomass and metabolite yield in microbial fermentation processes. The pH (X_3_) and temperature (X_4_) are known variables that impact microbial physiology and enzymatic activity. Because L-arginine is a precursor in the target compound's biosynthesis, its concentration (X_5_) was taken into consideration. Standard process parameters that have a major impact on microbial kinetics, oxygen transfer, and nutrient availability are inoculum size (X_6_), incubation time (X_7_), and agitation speed (X₈) were selected, and their high and low levels are shown in Table 1.

**Table 1:** High and low levels of variables used in Plackett-Burman design.

No.	Factors with their code	Low level (−1)	High level (+1)
1	Peptone (X_1_)	1 % (w/v)	5 % (w/v)
2	Maltose (X_2_)	1 %(w/v)	5 %(w/v)
3	pH (X_3_)	5	9
4	Temperature (X_4_)	25 °C	50 °C
5	L-arginine concentration (X_5_)	0.5 (w/v)	3 (w/v)
6	Inoculum size (X_6_)	1 % (w/v)	5 (w/v)
7	Incubation period (X_7_)	24 h	120 h
8	Agitation (X_8_)	50 rpm	200 rpm

The responses of 16 runs from the PBD experiments were utilized for generating regression coefficient values, and the significant variables were further optimized by response surface methodology (RSM).

The PBD was based on the first-order model with the following equation:

(1) Y=bo+∑bixi

Where Y is the response (L-arginase activity), b_o_ is the intercept, b_i_ is the linear coefficient, and x_i_ is the independent variable. Regression analysis and Pareto charts were used to identify critical components with a significant p-value (<0.05) as factors influencing the production of arginase. The experiments were developed using Design-Expert Version 13 software.

#### 2.5.2 Optimization of significant variables by response surface Methodology (RSM)

The significant variables screened through the PBD technique were subjected to a central composite design (CCD) (Design-Expert version 13 software), a popular second-order experimental design for developing sequential experimentation and predicting the levels of factors to get an optimal response through regression analysis. The statistical optimization strategy was applied for three significant factors: L-arginine concentration, incubation temperature, and peptone with five levels (Table 2).

**Table 2:** The actual values for the factors in CCD.

Factor	Code	Levels				
		−α	−1	0	1	+α
L-Arginine (%w/v)	A	−0.35	0.5	1.75	3	3.85
Peptone (% w/v)	B	−0.36	1	3	5	6.36
Temperature (^o^C)	C	16.47	25	37.5	50	58.5

Note: Negative values at the −α level are a result of statistical coding in CCD. In actual experiments, these concentrations were set to zero, as negative concentrations are not physically possible.

The second-degree polynomial equation was used to determine the relationship between the independent variables and the response. The results were used to fit the second-order polynomial model as expressed by the following equation:

(2) Y = β_0_ + β_1_A + β_2_B + β_3_C + β_12_AB + β_13_AC + β_23_BC + β_11_A ^2^ + β_22_B ^2^ + β_33_C ^2^

where Y is the predicted response (L-arginase activity); β_0_ is the intercept in the design; β_1_, β_2_ and β_3_ are the linear coefficients in the design; β_12_, β_13_ and β_23_ are the interaction coefficients in the design; β_11_, β_22_ and β_33_ are the squared (quadratic) coefficients in the design and A, B, and C are independent variables (as represented in Table 2).

### 2.6 Data analysis

Analysis of variance (ANOVA) was performed to analyze the variables and responses. A probability value criterion of less than 0.05 was used as a parameter for statistical significance. The polynomial model was expressed by the correlation coefficient (R^2^) associated with probability (P). Quadratic models for each graph were plotted as 3D response surface diagrams, and 2D contour plots which were generated using Design-Expert version 13 software.

The experimental model was validated by comparing the actual values obtained from the fermentation process performed using predicted media components to the predicted results by the design.

## 3 Result

### 3.1 Strain isolation and identification

Bacterial isolates that produced a pink color change on the MAA medium were selected, indicating L-arginase activity. Among these, strain BC2 exhibited the highest L-arginase production as confirmed by quantitative enzymatic analysis.

### 3.2 Screening and quantitative analysis of L-arginase production

**Fig. 1:**
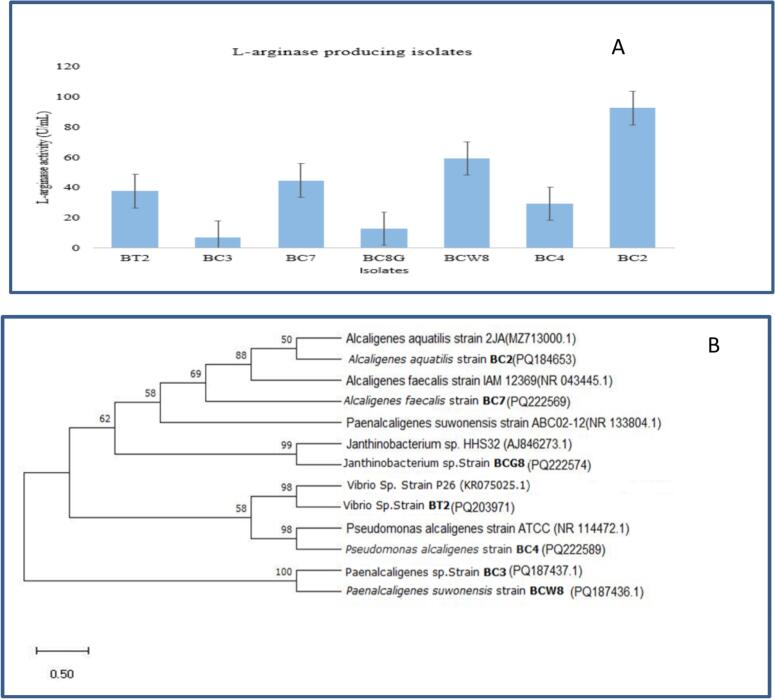
(A). Bar graph showing the L-arginase activity (U/mL) of different bacterial isolates (BT2, BC3, BC7, BC8G, BCW8, BC4, and BC2). Each bar represents the mean L-arginase activity for the corresponding isolate, with error bars indicating the standard deviation of replicate measurements. The isolate BC2 exhibited the highest L-arginase activity, while BC3 and BC8G showed the lowest activity among the tested isolates. (B).The tree was constructed using the Neighbor-Joining method, with evolutionary distances computed according to the Kimura 2-parameter model. The presented topology represents a consensus tree derived from 1,000 bootstrap replicates; bootstrap values (expressed as percentages) are indicated at the branch nodes to reflect the robustness of each clade. Strains labeled in bold (BC2, BC3, BC7, BC8, and BT2) represent isolates obtained in this study. Among them, all BC strains were screened for L-arginase production, with quantitative results provided Fig. 1A. The GenBank accession numbers for each sequence are shown in parentheses.

Bacterial isolates that converted the MAA medium to pink were initially screened for L-arginase activity. Those bacteria which hydrolyze L-arginine cause pH change and turn the medium from yellow to pink. To quantitatively assess enzyme production, we performed a colorimetric assay measuring urea formation following the method described by. Briefly, bacterial cultures were incubated under standardized conditions, and enzyme activity was measured by diacetyl monoxime method with the absorbance at 520 nm. Among all isolates tested, strain BC2 exhibited the highest L-arginase activity, with an average activity of 92.46 ± 0.19 units/mL, significantly higher than other isolates (p < 0.05).^27^ These quantitative results are summarized in Fig. 1A.

To identify isolate BC2, we extracted and amplified its genomic DNA using two universal primers. The PCR product of the isolate's 16S rRNA gene was then sequenced. The BLAST analysis result obtained from the NCBI database using BLASTN revealed that isolate BC2 has 97.30 % homology to *A. aquatilis* strain 2JA. Therefore isolate BC2 was identified as *A. aquatilis* based on 16S rRNA gene sequence analysis and deposited at GenBank by the accession number of PQ184653.^27^ Phylogenetic analysis was performed using MEGA 13 software via the neighborhood-joining method. The maximum likelihood and Kimura 2-parameter model with 1000 bootstrap replications were employed to estimate the tree topology.^28^ Phylogenetic analysis confirmed the placement of isolate BC2 within the Alcaligenes genus. Isolate BC2 and strain 2JA formed a distinct clade on the phylogenetic tree (Fig. 1B), indicating their close evolutionary relationship.

Our investigation revealed that isolate BC2, *A. aquatilis* BC2, is the most effective strain of bacteria for L-arginase production compared to other isolates. Notably, it also produces L-arginase, a capability that has not been previously investigated; thus, we have chosen it for further media optimization.

#### 3.2.1 Production of L-arginase

Alcaligenes aquatilis BC2, which was isolated from Lake Chitu, a soda lake in Ethiopia is identified as the highest producer of L-arginase, exhibiting an activity level of 92.45 U/mL. The physicochemical and nutritional parameters were initially investigated by OFAT for the production of L-arginase. In this method one factor is varied at once while the other factors are maintained constant. The effects incubation period (24 h, 48 h, 72 h, 96 h and 120 h) was optimized for the production of L-arginase. The highest L-arginase activity was shown at 24 h incubation period (Table 3). The effect of incubation temperature (25–50 °C with the interval of 5 °C) for optimum L-arginase activity has been investigated. High L-arginase activity were recorded at 30 °C (Table 3).

**Table 3:** Initial optimization of L-arginase producing microbes by one factor at-a- time (OFAT).

Isolate	C-source% (w/v)		N-source% (w/v)	Arginine conc. (w/v)	pH	T^o^ (^o^ C)	Incubation time (h)	Agitation speed (rpm)	Inoc. Vol% (v/v)	Reference
*L. acidophilus*	Sucrose		Yeast extract	15 mM	6	40	24 h	−	1 %	^31^
*Alcaligenes faecalis* B26	Lactose		Yeast extract	6 %	7.5	35	60 h	150	0.2 ml	^32^
*Alcaligenes faecalis* Strain ZB	-		-		7	35	-	200	-	^33^
*Pseudomonas* sp. strain PV1	Maltose		Yeast extract	1 %	8.5	37	24 h	150	10 ml	^34^
*Bacillus licheniformis* OF2	Maltose		Peptone	1 %	8	35	48 h	−	−	^35^
*Idiomarina sediminium* H1695	Maltose		Casein	2 %	9	37	120 h	−	10 %	^36^
*Aspergillus niger* AUMC16187	Dextrose		NH_4_Cl	-	7	27	168 h	−	-	^37^
***A. aquatilis* BC2**	**Maltose**		**Peptone**	**2%**	**9**	**30**	**24 h**	**150**	**3 %**	**The present study**

The pH of the production medium influences enzyme activity by affecting the solubility and membrane transport of nutrients, altering the ionic state of substrates, and impacting enzyme stability.^29^ The maximum L-arginase production was observed at pH of 9 (Table 3). To study the effect of carbon source on L-arginase production, maltose, lactose, glucose, galactose, and sucrose were used. The highest enzyme was produced in maltose (1 % w/v).

For nitrogen sources different organic (peptone, tryptone, and yeast extract) and inorganic sources (NH_4_Cl and NaNO_3_) were used. The highest levels of L-arginase were seen while peptone, an organic nitrogen source, was used (Table 3). The effect of substrate concentration was optimized with different concentrations (0.5 to 3 % w/v) of L-arginine. In the present study, the optimum L-arginase was recorded at 2 % of L-arginine concentration.

Agitation speed is quite important in increasing bacterial growth and enzyme production. This is accomplished by assisting oxygen transfer and guaranteeing that microbial cells get enough nutrients.^30^ Different agitation speeds (100, 150, 200, and 250 rpm) were used for optimization. The highest activity was observed at 150 rpm. In the present study, the optimum L-arginase was observed at 3 % v/v inoculum size (Table 3).

### 3.3 Identification of significant variables by PBD

The impact of eight factors on the production of L-arginase by *Alcaligenes aquatilis* BC2 was examined over 16 experimental runs using a Plackett-Burman design (PBD). Table 4 presents the PBD setup, including the selected variables and their corresponding responses for L-arginase production. The experimental results showed variability, with L-arginase activity ranging between 61.88 and 189.05 U/mL. The highest L-arginase activity was observed in run 6 (189.05 U/mL), and the lowest L-arginase was recorded in run 5 (61.88 U/mL).

**Table 4:** Experimental Design Matrix for Plackett-Burman Screening of Variables Affecting L-Arginase Production by *Alcaligenes aquatilis* BC2.

		Factor 1	Factor 2	Factor3	Factor 4	Factor 5	Factor 6	Factor 7	Factor 8	Response	
	Run	A:Peptone	B: Maltose	C:pH	D:Temp	E:Arginine	F:Inoculum size	G:Incubation period	H:Agitation	L arginase activity (U/mL)	
		% (w/v)	% (w/v)		^0^C	% (w/v)	% (v/v)	Hour	(rpm)	Experimental	Predicted
	1	−1	−1	1	−1	1	1	1	−1	145.93 ± 1.76	146.66
	2	−1	1	−1	−1	1	1	−1	1	119.16 ± 1.38	119.89
	3	1	−1	1	−1	−1	1	−1	1	109.29 ± 1.24	109.1
	4	−1	−1	1	1	1	−1	−1	1	133.03 ± 0.62	132.84
	5	−1	1	1	1	−1	1	−1	−1	61.88 ± 1.46	61.15
	6	1	1	1	−1	1	−1	−1	−1	189.05 ± 0.91	188.32
	7	1	−1	−1	1	1	1	−1	−1	170.05 ± 2.56	170.24
	8	−1	−1	−1	1	−1	1	1	1	160.43 ± 0.33	159.7
	9	1	1	1	1	1	1	1	1	137.91 ± 0.1	138.1
	10	1	−1	1	1	−1	−1	1	−1	130.6 ± 1.95	131.33
	11	−1	−1	−1	−1	−1	−1	−1	−1	127.52 ± 0.37	127.71
	12	−1	1	−1	1	1	−1	1	−1	152.09 ± 2.23	151.90
	13	1	1	−1	1	−1	−1	−1	1	177.08 ± 1.6	177.81
	14	1	−1	−1	−1	1	−1	1	1	167.22 ± 0.41	166.49
	15	1	1	−1	−1	−1	1	1	−1	118.01 ± 0.71	117.82
	16	−1	1	1	−1	−1	−1	1	1	75.4 ± 2.87	75.59

The actual values corresponding to the coded levels (−1 and + 1) for each variable are provided in Table 1. Each result of experimental data represents the mean ± standard deviation of three independent experiments performed in triplicate.

Based on the analysis of variance (ANOVA), the most significant factors affecting L-arginase production were identified as L-arginine (E), temperature (D), and peptone (A), as shown in Table 5. A first-order polynomial equation was developed to express L-arginase production as a function of these independent variables:

Y = 135.92 + 13.99A + 4.47D + 15.89E

where, Y represents the response (L-arginase activity), while A, D, and E correspond to peptone, temperature, and L-arginine concentration, respectively, with their respective coefficients indicating the magnitude of their influence.

**Table 5:** Statistical analysis of PBD.

Source	SS	Df	MS	F-value	P- value	Coefficient
Model	18185.97	13	1398.92	619.54	0.0016	135.92
A-Peptone	3129.56	1	3129.56	1386.00	0.0007*	13.99
B-Maltose	805.00	1	805.00	356.51	0.0028	−7.09
C-pH	2716.23	1	2716.23	1202.95	0.0008	−13.03
D-Temp	319.43	1	319.43	141.47	0.0070*	4.47
E-Arginine	4039.56	1	4039.56	1789.01	0.0006*	15.89
F-Inoculum size	1045.39	1	1045.39	462.98	0.0022	−8.08
G-Incubation	0.0176	1	0.0176	0.0078	0.9378	0.03
H-Agitation	15.23	1	15.23	6.74	0.1218	−8.0
Residual	4.52	2	2.26			
Cor Total	18190.49	15				

MS- Mean Square, df- degree of freedom, SS- Sum of Squares. R^2^ = 0.9998, Adjusted R^2^ = 0.9981, Predicted R^2^ = 0.9841 and Adequate precision = 90.4733.

**Fig. 2:**
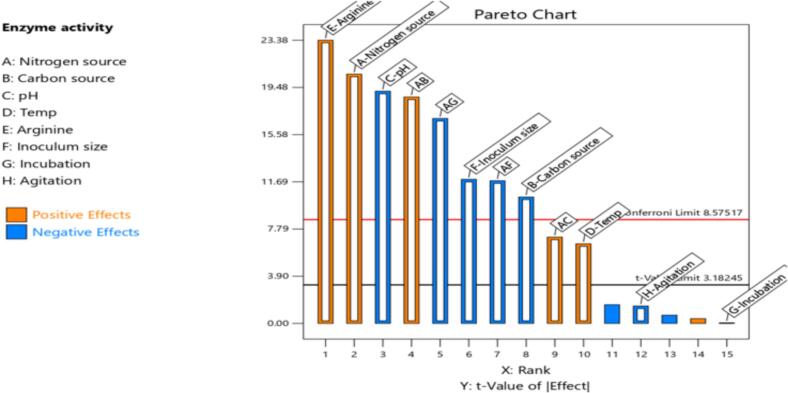
Pareto chart demonstrating the standardized effects of eight factors on enzyme activity (A: source of nitrogen, B: source of carbon, C: pH, D: temperature, E: arginine, F: size of inoculum, G: incubation, H: agitation). Each bar's length indicates the effect's magnitude; blue bars show a negative effect, and orange bars show a positive effect. Factors extending beyond the Bonferroni limit (red line) are considered statistically significant. (For interpretation of the references to color in this figure legend, the reader is referred to the web version of this article.)

The Pareto chart (Fig. 2) shows the significant experimental parameters influencing enzyme production. It illustrates both the positive and negative effects of individual factors and their combinations. Based on the chart, arginine, peptone, and temperature exhibited a positive impact on L-arginase production. Statistical analysis of the Plackett-Burman design (PBD) revealed that the model F-value of 619.54 confirms its significance. A p-value of less than 0.05 was considered statistically significant. In this study, the variables A, B, C, D, E, and F were identified as significant model terms (Table 5). Based on the statistical analysis of the Plackett-Burman Design (Table 5) and the corresponding Pareto chart (Fig. 2), peptone, arginine concentration, and incubation temperature were selected for further optimization. These three factors exhibited the highest F-values, statistically significant P-values (p < 0.05), and positive coefficients, indicating a strong and beneficial impact on L-arginase production. Specifically, the positive coefficients for peptone, arginine, and temperature demonstrate that increasing these variables enhances enzyme yield.

In contrast, maltose, pH, and inoculum size, while statistically significant, showed negative coefficients, suggesting that higher levels of these factors adversely affect enzyme production. Therefore, these variables were not prioritized for further optimization. This selection strategy is supported by both the magnitude and direction of the effects observed, ensuring that subsequent optimization efforts focus on the most influential and beneficial factors.

The statistical analysis revealed a strong model fit, with an R^2^ value of 0.9998 (Table 5), indicating that 99.98 % of the variability in L-arginase production could be explained by the selected factors. A high R^2^ value suggests that the independent variables included in the model have a very strong predictive capability for the response.^38^

The adjusted R^2^ accounts for the number of predictors in the model. Its high value (close to the R^2^) confirms that the model is not overfitted and remains robust even after adjusting for additional terms.^39^ In the present model, a predicted R2 of 0.9841 (Table 5) is very close to the adjusted R2 (0.9981), indicating excellent agreement between predicted and actual values and validating the model's reliability. Additionally, the adequate precision ratio of the present model, which is 90.4733 (Table 5), emphasizes the ability of the model to differentiate between significant effects and background noise, hence making it highly appropriate for optimization purposes. Desirable is generally considered greater than 4, and a value as great as 90.4733 suggests an exceptionally strong signal. This implies the model is quite reliable for further research and optimization.^40^

ANOVA results, supported by a high F-value of 619.54 (p < 0.05), confirmed the model's statistical significance. Among the investigated factors, arginine, peptone, and temperature emerged as the most influential, with positive coefficients indicating their favorable impact on enzyme production. They were selected for further optimization by CCD-RSM.

### 3.4 Optimization with central composite design (CCD)

Response surface methodology (RSM) is a popular statistical technique for maximizing cultural conditions during the fermentation of several enzymes. This strategy has been used by several scientists to maximize enzyme output from microorganisms.

Three factors affecting L-arginase production were found to be significant: L-arginine, peptone, and temperature, based on the placket burman design (PBD) output.

Further optimization of these variables was done using RSM-CCD.

Twenty experimental runs were carried out using several permutations of the three variables given in Table 6. Other fixed ingredients of fermentation; pH 9, 1 % (w/v) maltose, 24 h incubation time, 150 rpm agitation, 0.5 % (w/v) K_2_HPO_4_, and 3 % (v/v) inoculum volume were kept constant across all experimental runs. The experimental results were used to create a predictive model offering estimated as well as actual (observed) values of L-arginase activity.

**Table 6:** Experimental setup of central composite design.

Run	Factor 1	Factor 2	Factor 3	L-arginase activity	
	A:arginine (% w/v)	B:Peptone (% w/v)	C:temperature (^o^C)	Actual (U/mL)	Predicted (U/mL)
1	3	5	50	209.77 ± 1.18	214.99
2	1.75	3	37.5	281.14 ± 1.03	287.23
3	−0.35	3	37.5	121.03 ± 3.66	125.83
4	1.75	3	37.5	288.15 ± 0.55	287.23
5	1.75	3	37.5	288.15 ± 0.19	287.23
6	1.75	3	37.5	288.64 ± 0.65	287.23
7	1.75	3	16.5	139.15 ± 1.95	139.59
8	3	5	25	160 ± 0.91	164.32
9	1.75	3	37.5	288.79 ± 0.87	287.23
10	0.5	1	25	142.09 ± 0.16	138.46
11	0.5	5	50	221.8 ± 1.06	222.45
12	1.75	3	58.5	210.26 ± 2.5	207.56
13	3	1	50	128.18 ± 0.07	130.85
14	1.75	6.36	37.5	263.75 ± 1.07	259.01
15	1.75	3	37.5	288.15 ± 0.03	287.23
16	0.5	1	50	171.35 ± 0.51	168.62
17	1.75	−0.36	37.5	181.03 ± 0.89	183.42
18	3.85	3	37.5	117.95 ± 0.62	110.89
19	3	1	25	127.22 ± 0.04	128.16
20	0.5	5	25	145.4 ± 0.73	144.32

The maximum L-arginase (288.79 ± 0.87 U/mL) was observed in run 9, and the minimum L-arginase (117.95 ± 0.62) was recorded at run 18 (Table 6). The result was presented in mean ± SD.

The data obtained from the experiments were analyzed using Analysis of Variance (ANOVA) to determine the significance of the factors and their interactions. Additionally, a second-order polynomial equation was developed to describe how L-arginase production depends on the independent variables.

The second-order polynomial equation for predicting L-arginase activity is as follows:

Y = 287.23 − 4.44A + 22.50B + 20.21C + 7.58AB − 6.87AC + 11.99BC − 59.70 A^2^ − 23.32B^2^ − 40.18C^2^.

Where Y represents the response, which is the activity of L-arginase (in U/mL), A, B, and C correspond to the concentration of L-arginine, concentration of peptone, and temperature, respectively.

This equation models the relationship between the independent variables (A, B, and C) and the response (Y), incorporating both linear, interaction, and quadratic effects to optimize L-arginase production.

The analysis of variance (ANOVA) results shown in Table 7 demonstrate the model's significance, as indicated by a Fisher’s F-test value of 420.28. High F-value demonstrates that the model effectively explains a significant proportion of the variability in the response variable and confirms that the factors included in the model have a statistically significant impact on the response.^41^ Furthermore, the confidence level of 99.9 % (p < 0.0001) shows the robustness of the model, indicating that it accurately represents the relationship between the independent variables (A-arginine concentration, B-N-source, C-temperature) and the dependent variable (L-arginase activity). For a model to be statistically significant The F-value should be higher and the p-value should be lower.^40^ Based on the results obtained, the designed model was determined to be statistically significant for the production of L-arginase.

**Table 7:** ANOVA table for CCD quadratic model.

Source	SS	df	M S	F-value	p-value	
**Model**	86197.26	9	9577.47	420.28	<0.0001	*
A-arginine	269.35	1	269.35	11.82	0.0064	*
B-Peptone	6912.37	1	6912.37	303.33	<0.0001	*
C-temperature	5577.14	1	5577.14	244.74	<0.0001	*
AB	459.20	1	459.20	20.15	0.0012	*
AC	377.16	1	377.16	16.55	0.0023	*
BC	1150.80	1	1150.80	50.50	<0.0001	*
A^b^	51371.20	1	51371.20	2254.29	<0.0001	*
B^b^	7839.90	1	7839.90	344.03	<0.0001	*
C^b^	23269.80	1	23269.80	1021.13	<0.0001	*
**Residual**	227.88	10	22.79			
Lack of Fit	183.85	5	36.77	4.18	0.0714	NS
Pure Error	44.03	5	8.81			
**Cor Total**	86425.14	19				

* - significant, NS- non significant SS- Sum of Squares, MS- Mean Square, Df- degree of freedom, R^2^ = 0.9974, Adjusted R^2^ = 0.9950, Predicted R^2^ = 0.9831 and Adequate precision = 52.2407.

To evaluate the goodness-of-fit of the model, several statistical measures were analyzed, including R^2^ (coefficient of determination), adjusted R^2^, predicted R^2^, and adequate precision. The results demonstrate that the designed model is highly reliable and effective for predicting L-arginase activity.

The model's R2 value is 0.9974, indicating that 99.74 percent of the change in L-arginase activity is explained by the model (Table 7). The R2 value varies between 0 and 1; above 0.75 indicates the model fits the data well.^42^ Furthermore, the adjusted R^2^ value is 0.9950, which takes the quantity of predictors in the model into account. Even after adjusting for model complexity, 99.50 % of the variability is still explained, underscoring the robustness of the model.

The predicted R^2^ has a value of 0.9831, which is close to the adjusted R^2^. This resemblance shows the model does not overfit and has good predictive ability for new data. The adequate precision value is 52.2407, significantly above the recommended value of 4. This high value confirms that the model has a sufficient signal-to-noise ratio, enabling it to effectively navigate and optimize the design space.^43^

Lack of fit has an F-value of 4.18 and a p-value of 0.0714 (Table 7). The lack of fit is not statistically significant since the p-value is above 0.05. For a model to be appropriate, the lack of fit test values should not be significant.^44^ This suggests that our model adequately fits the data, and any deviations from the model can be attributed to random error rather than an inadequate model structure. In view of Table 7, the concentration of peptone had the strongest influence on the L-arginase production, with an F-value of 303.33, followed by incubation temperature F-value of 244.74.

The predicted vs. actual plot demonstrates that the model is highly effective in predicting enzyme activity (Fig. 3A). The close alignment of points to the diagonal line, combined with the consistent color-coded distribution, confirms that the model has a strong correlation between predicted and actual values.^45^ It also exhibits high accuracy across the range of enzyme activities.

**Fig. 3A:**
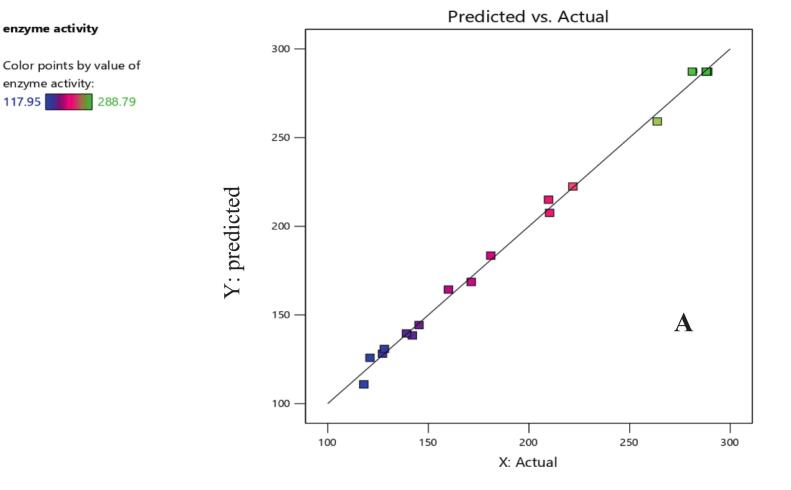
Predicted versus actual plot illustrating the performance of the model in predicting enzyme activity. The close clustering of data points around the diagonal line suggests a good correlation between the predicted and actual values. The color gradient indicates the distribution of enzyme activity across the data points, with lower activities shown in blue/purple and higher activities in green/yellow. (For interpretation of the references to color in this figure legend, the reader is referred to the web version of this article.)

The model fitness may also be interpreted using the residual graph, which offers helpful information (Fig. 3B). For the residuals to be normally distributed, data points should closely follow the line.^46^

**Fig. 3B:**
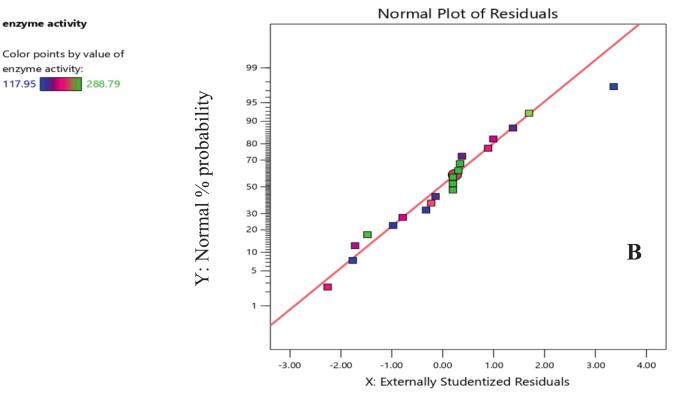
Normal probability plot of the externally studentized residuals for enzyme activity. The externally studentized residuals are plotted against their expected normal distribution. The color gradient indicates the enzyme activity associated with each residual, with lower values in blue/purple and higher values in green/yellow. The closer the points follow the straight line, the better the normality assumption is met. (For interpretation of the references to color in this figure legend, the reader is referred to the web version of this article.)

In the current study, the model's normality is a crucial premise for regression analysis which is indicated by the points' adherence to the line. This demonstrates the model's suitability and validity. The model is functioning well, as seen by the residuals vs. predicted plot (Fig. 3C), which displays a random distribution of residuals around zero with no discernible trends or critical assumption violations. This shows that the model fits the data well and reflects the underlying correlations between the variables and enzyme activity. The consistency and dependability of the model are further supported by the lack of outliers.^47^

**Fig. 3C:**
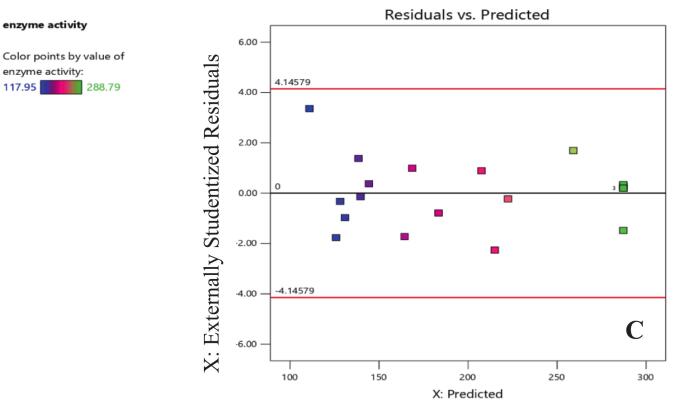
Residuals versus predicted values for the enzyme activity model. Residual plot used to assess the variance and linearity of the enzyme activity model. The externally studentized residuals are plotted against the predicted enzyme activity values. Each point is colored according to its enzyme activity value, as shown in the legend. The horizontal line at zero highlights the expected distribution of residuals, and the red lines represents the identification of potential outliers. (For interpretation of the references to color in this figure legend, the reader is referred to the web version of this article.)

The 2D contour and 3D response surface plots of the regression equation were created by two independent variables with their effect on L-arginase production, while the third variable was maintained at its central level. It offers a visual representation of how experimental values interact with the responses predicted by the model for individual variables.^48^ These graphical tools are instruments in identifying the optimal levels of these variables to maximize L-arginase production.

The shape of the contour plots provides insights into the strength and nature of interactions between the corresponding variables, as noted by Sharma and Sharma..^49^ Specifically, elliptical contour plots suggest significant interactions among the variables, while circular contour plots indicate that there are no substantial interactions between them.^50^

The 2D contour plots and their respective 3D response surface plots presented in Fig. 4A and 4B show the interaction between peptone and L-arginine at the middle value of incubation temperature. As depicted in Fig. 4A and the ANOVA table 7, the nearly elliptical shape of the contour plot suggests that there is an interaction between the peptone and arginine concentrations (p < 0.0012) in their effect on L-arginase production. The bell-shaped response curve (Fig. 4B) suggests moderate L-arginine and peptone ensure optimal L-arginase production. A medium level of peptone supplies the necessary nutrients without diverting metabolic resources. However, excessive peptone can result in the buildup of toxic byproducts, such as ammonia, during microbial metabolism. This accumulation can inhibit both enzyme production and microbial growth, as noted by Sanchez and Demain.^51^

**Fig. 4A:**
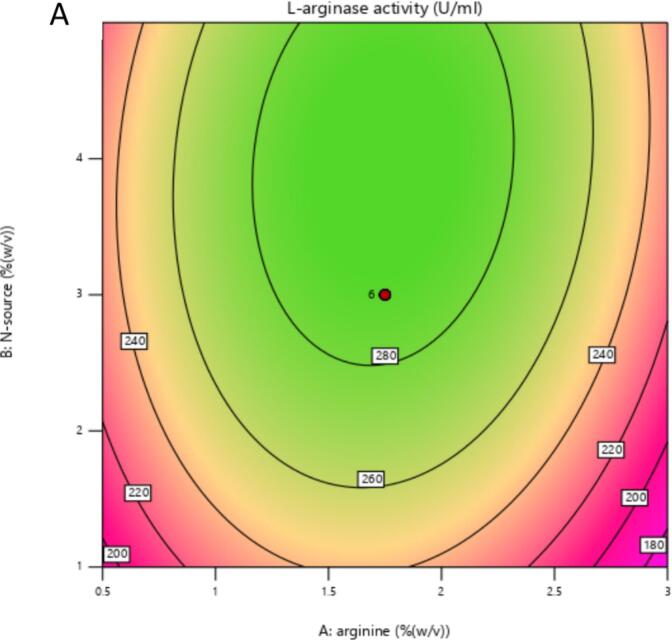
Contour plot illustrating the effect of Arginine (% w/v) and Nitrogen source (% w/v) concentrations on L-arginase activity (U/mL). Higher activity seen in the green areas shows the enzyme activity levels as indicated by the color gradient and labeled contour lines. The red dot denotes the expected best mix of Arginine and Nitrogen source for maximizing L-arginase activity inside the examined experimental area.

**Fig. 4B:**
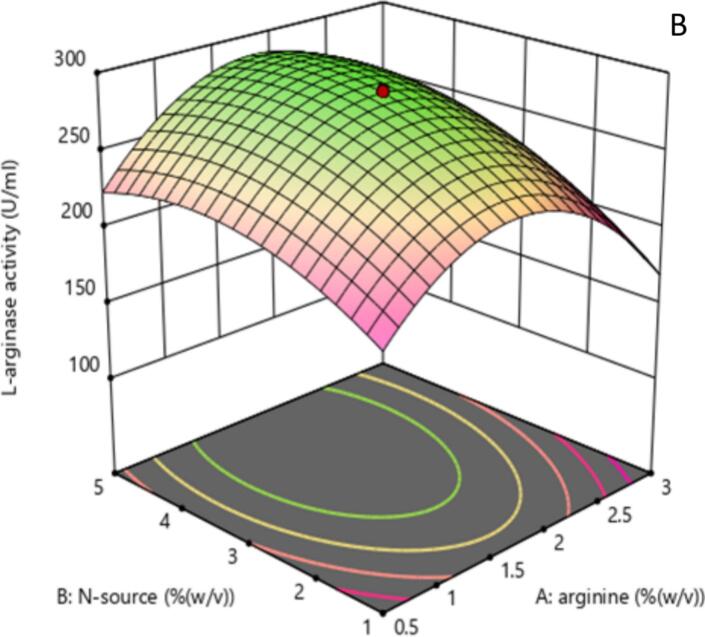
3D response surface showing the interactive influences of Arginine concentration (A: % w/v) and Nitrogen source concentration (B: % w/v) on L-arginase activity (U/mL). With higher and greener areas suggesting more activity, the surface's height and color show the enzyme activity levels. The contour lines projected onto the base represent lines of constant L-arginase activity. The red dot indicates the predicted maximum response within the experimental design space.

The contour and response surface plots examining the interactions between arginine and temperature (Fig. 5A, Fig. 5B) as well as temperature and peptone (Fig. 5C, Fig. 5D) revealed elliptical shapes in the contour plots and parabolic curvatures in the 3D plot. This observation suggests that these interactions play a significant role in influencing L-arginase production. Contour plots generated using RSM often show bell-shaped responses, where intermediate levels of input variables produce maximum output.^52^ As illustrated in Fig. 5A, Fig. 5B, the production of L-arginase is influenced by the concentration of L-arginine, exhibiting a parabolic response across varying temperature levels. The highest yield of L-arginase was observed when the L-arginine concentration was in the range of 1.5–2 %. In this study, it was noted that L-arginase production increased as both temperature and L-arginine concentration were raised to their optimal levels. These findings indicate a strong interaction between L-arginine concentration and temperature in the production of L-arginase by *Alcaligenes aquatilis* BC2, with a statistically significant p-value of 0.0023 (Table 7).

**Fig. 5A:**
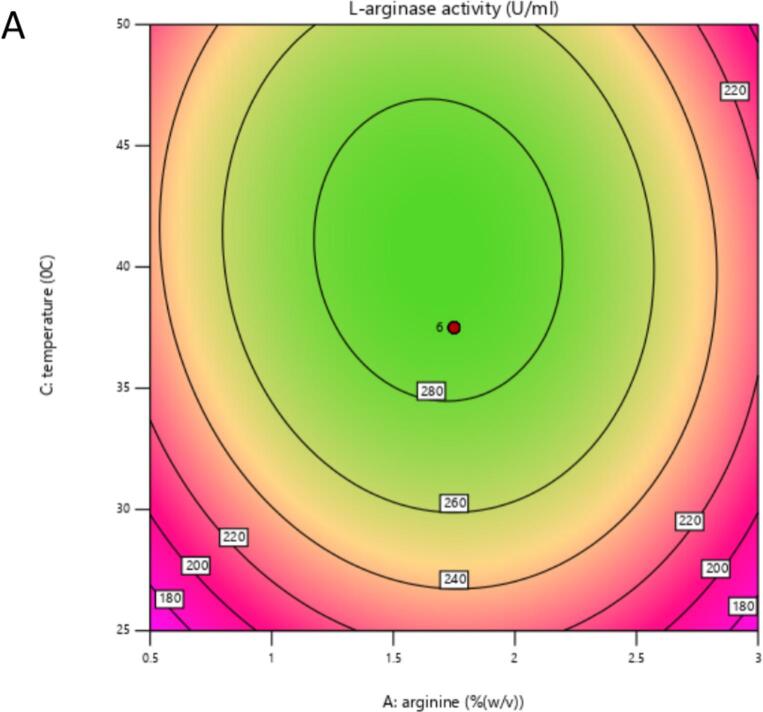
Contour plot showing the interactive effects of Arginine concentration (A: % w/v) and Temperature (C: °C) on L-arginase activity (U/mL). The plot indicates that higher enzyme activity shown by green areas and rising contour values. The red dot identifies the expected Arginine and Temperature levels that produce the maximum L-arginase activity inside the examined ranges.

**Fig. 5B:**
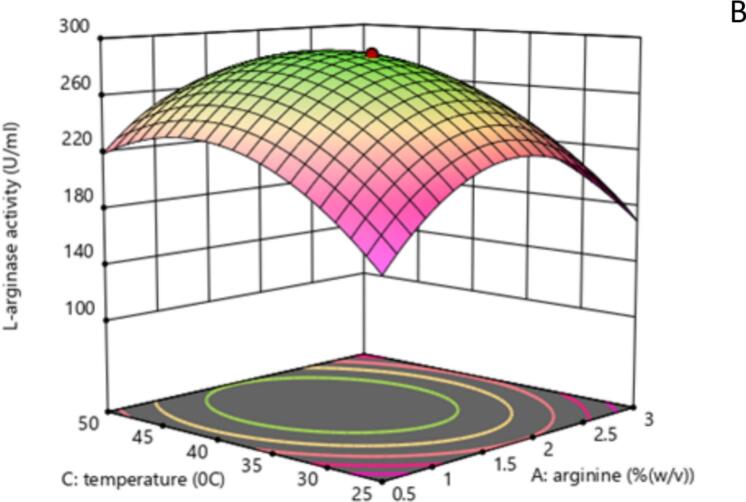
Response surface plot showing the ideal Arginine concentration and Temperature conditions to maximize L-arginase activity. Indicated by the highest point on the green surface and the position of the red dot, the graph shows a peak enzyme activity at roughly 1.75 % (w/v) Arginine and approximately 37–38 °C.

Similarly, the response behavior was also analyzed between peptone and temperature with the central value of the other factor (arginine concentration). The production of L-arginase was affected by peptone concentration and followed a parabolic curve. Extreme conditions of temperature and peptone decreased the production of the enzyme, and the optimum production of L-arginase was recorded in the range of 2.5–3 % peptone concentration and 35–40 °C of temperature (Fig. 5C, Fig. 5D).

**Fig. 5C:**
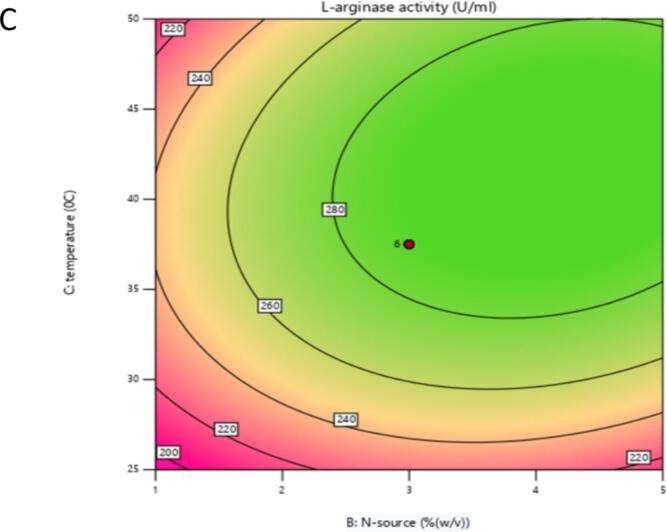
Shows a contour plot of the influence of nitrogen supply concentration (%w/v) and temperature (°C) on L-arginase activity (U/mL). The plot demonstrates that increased enzyme activity (represented by green regions and higher contour values) is obtained at certain combinations of the two parameters, implying an ideal temperature range of 37–38 °C and a nitrogen supply concentration of roughly 3 % (w/v). The red dot indicates the projected amounts of nitrogen supply and temperature that provide the maximum L-arginase activity within the examined ranges.

**Fig. 5D:**
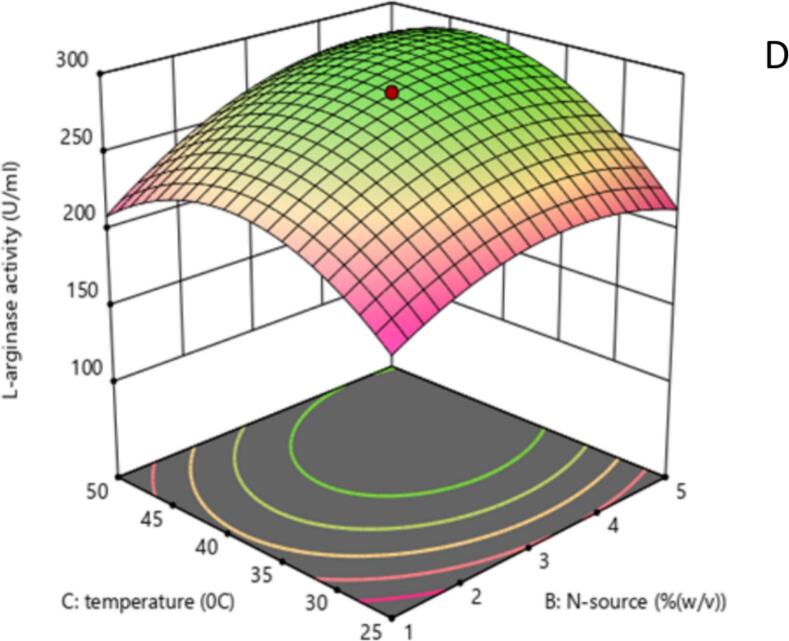
Response surface plot illustrating the effect of Nitrogen source concentration (% w/v) and Temperature (°C) on L-arginase activity (U/mL). The surface color and height represent the enzyme activity level, with higher activity indicated by the green and elevated regions. The red dot marks the predicted optimal combination of Nitrogen source concentration and Temperature for maximizing L-arginase activity within the tested ranges.

### 3.5 Validation of the experimental model

The validation process was carried out under the conditions anticipated by the experimental model. The model predicted optimal levels of 1.75 % w/v L-arginine, 3 % w/v peptone, and an incubation temperature of 37.5 °C, tested in triplicate. Under these ideal conditions, the production of L-arginase reached 288.79 U/mL, which closely matched the model's predicted response of 287.23 U/mL. This verification demonstrated a high level of model accuracy at 99.46 %. Additionally, it was observed that optimizing the medium using statistical design resulted in a 3.1-fold increase in yield.

## 4 Discussion

This study is the first to report the production of L-arginase enzyme from the bacteria isolated from Soda Lake of Ethiopia. The L-arginase producing bacteria were screened quantitatively based on their L-arginase activity as shown in Fig. 1A, isolate BC2 was observed as having higher L-arginase activity. The identification of isolate BC2 as *A. aquatilis* based on 16S rRNA gene sequencing aligns with previous studies that have reported *Alcaligenes* species as producers of L-arginase.^32^

The phylogenetic tree constructed using MEGA 13 software grouped isolate BC2 closely with *A. aquatilis* strain 2JA, reinforcing their genetic relatedness and supporting the classification of BC2 within the Alcaligenes genus.

Various nutritional and physicochemical parameters influence L-arginase production by microorganisms. In the present study the highest L-arginase production was reported at a temperature of 30 °C (Table 3), which is consistent with reports by Agbaje et al., Selim et al. and Ibrahimi et al.^32^, ^33^, ^35^ under optimal production around 35 °C. The decline in enzyme activity beyond this optimal temperature is likely due to enzyme denaturation, which alters its structure and impairs the active site.

Optimization of pH revealed maximum L-arginase production at pH 9, indicating the alkaline nature of the isolate. This finding is in agreement with Unissa et al..^36^ Regarding incubation period, the optimum enzyme activity was recorded at 24 h, consistent with studies by Gutam et al. and Nadaf et al..^31^, ^34^ The decrease in activity with longer incubation may be attributed to nutrient depletion.

The type and concentration of carbon source affects L-arginase production by *A. aquatilis* BC2. Maltose at 1 % (w/v) yielded the highest enzyme production, suggesting it is a favorable carbon source, possibly due to its non-repressive and energy-efficient properties. This aligns with findings reported in.^34^, ^35^, ^36^ In contrast, other studies have reported sucrose,^31^ lactose,^32^ and dextrose^37^ as optimal carbon sources (Table 3). Among different nitrogen sources used, the organic nitrogen source, peptone showed highest L-arginase production. Other investigations have also indicated that the addition of peptone to production conditions enhances the synthesis of L-arginase enzymes, including *Bacillus lechiniformis*.^35^ Metabolism of inorganic nitrogen often releases acidic byproducts, lowering culture pH and potentially inhibiting enzyme stability. Other studies with *L. acidophilus, Alcaligenes faecalis* B26, and *Pseudomonas* sp. strain PV1^31^, ^32^, ^34^ yeast extract has been reported as the best nitrogen source for producing L-arginase.

In our study 2 % arginine concentration was optimum for the production of L-arginase by *A. aquatilis* BC2, consistent with Unissa et al..^36^ Higher L-arginine concentrations may act as corepressors or transcriptional suppressors, inhibiting arginase gene expression. Additionally, elevated substrate levels can increase osmotic pressure, causing stress that diverts bacterial resources from enzyme production to survival.^53^ Enzyme production decreases as the bacteria focus on survival mechanisms to counteract osmotic stress. In the present study, the highest activity was observed in the agitation speed of 150 rpm. Similar results were demonstrated by Agbaje et al. and Nadaf and Vedamurthy.^32^, ^34^ The decline of L-arginase production above 150 rpm may be due to shear forces killing the bacteria, and high agitation can sometimes cause enzymes to aggregate or clump together, reducing their effective concentration and accessibility to the substrate.

Single-factor optimization does not account for interactions between variables, which can lead to misinterpretation. Therefore, statistical optimization using Response Surface Methodology (RSM) was employed in this study. Plackett-Burman Design (PBD) identified L-arginine, peptone concentration, and incubation temperature as the most significant factors influencing L-arginase production. Central Composite Design (CCD) further optimized these variables and revealed significant interactions between peptone and incubation temperature (P < 0.0001), L-arginine and peptone concentration (P < 0.0012), and L-arginine and incubation temperature (P < 0.0023). Similar observations were reported by Abdelraof et al.,^54^ during optimization of L-arginase production by *Streptomyces diastaticus* MAM5. Gautam et al.,^31^ also highlighted the critical role of L-arginine concentration in optimizing L-arginase production by Lactobacillus acidophilus using RSM. These findings collectively emphasize the importance of optimizing both L-arginine concentration and temperature to achieve maximum L-arginase production.

Model evaluation showed close agreement between experimental and predicted values, indicating the effectiveness of the experimental design for process optimization. Under optimized conditions, L-arginase production increased significantly from 92 U/mL (non-optimized) to 288.79 U/mL, representing a 3.13-fold enhancement. Consistent with the findings of the present study, several researchers have demonstrated significant improvements in L-arginase production through the application of Response Surface Methodology (RSM). For instance, Guatim et al.^31^ reported enhanced optimization of L-arginase production by *L. acidophilus*. Similarly, Momin B et al.^55^ and Abdelorf et al.^54^ utilized statistical design approaches to optimize L-arginase production, with the former focusing on *Bacillus licheniformis* M09 and the latter on a Streptomyces isolate.

## 5 Conclusion

This study focuses on enhancing L-arginase production from A. aquatilis BC2, a bacterium isolated from an Ethiopian soda lake. While various bacteria are known to produce L-arginase, statistical optimization for maximizing its production in A. aquatilis BC2 has been limited. The present study addresses this gap by employing PBD and CCD within Response Surface Methodology to optimize L-arginase production. Initially, PBD was used to screen eight factors influencing L-arginase production. The most significant variables arginine concentration, peptone concentration, and incubation temperature were further optimized using CCD with 20 experimental runs to determine the interaction and optimal levels of these key factors. This statistical optimization strategy resulted in a significant improvement in L-arginase yield, increasing it from 92 U/mL to 288.79 U/mL, a 3.1-fold enhancement. The study demonstrates the effectiveness of statistical methods in optimizing L-arginase production by A. aquatilis BC2. The enhanced production of L-arginase achieved through statistical optimization not only facilitates cost-effective industrial-scale enzyme manufacturing but also advances the development of enzyme-based anticancer therapies by providing higher yields of this therapeutically important enzyme.

## 6 Consent for publication

All authors state that there are no conflicts of interest and that there are no other bodies to take into consideration for publication. The corresponding author, give my consent and on behalf of co-authors for the publication of contents and other details in this material to be published in the Journal.

## 7 Availability of data and materials

The manuscript and all required data are uploaded to the web system online. If additional information is needed, the corresponding author will provide it upon request.

## 8 Authors' contributions

Study conception and design: B Getie; B Andualem, Data collection and analysis: B Getie, Analysis and interpretation of results, editing and reviewing of the manuscript : B Getie; T Geremew; T Milkessa; K Ayalew; M Aemero; B Andualem.

## CRediT authorship contribution statement

**Birhan Getie Assega:** Writing – review & editing, Writing – original draft, Methodology, Formal analysis, Data curation, Conceptualization. **Kefyalew Ayalew Getahun:** Writing – review & editing, Resources, Funding acquisition, Data curation. **Tamene Milkessa Jiru:** Writing – review & editing, Project administration, Funding acquisition, Formal analysis. **Tsehayneh Geremew Yohannes:** Writing – review & editing, Supervision, Resources, Project administration. **Mulugeta Aemero:** Writing – review & editing, Validation, Supervision, Resources. **Berhanu Andualem:** Writing – review & editing, Supervision, Resources, Project administration, Methodology, Funding acquisition, Data curation, Conceptualization.

## Ethics approval and consent to participate

Not applicable.

## Funding

This research did not receive any specific grant from funding agencies in the public, commercial, or not for-profit sectors.

## Declaration of competing interest

The authors declare that they have no known competing financial interests or personal relationships that could have appeared to influence the work reported in this paper.

## References

[1] Liu B., Zhou H., Tan L., Siu K.T.H., Guan X.-Y. (2024). Exploring treatment options in cancer: tumor treatment strategies. Signal Transduct Target Ther.

[2] Xia Y., Sun M., Huang H., Jin W.-L. (2024). Drug repurposing for cancer therapy. Signal Transduct Target Ther.

[3] Hassan F.S., El-Fakharany E.M., El-Maradny Y.A. (2024). Comprehensive insight into exploring the potential of microbial enzymes in cancer therapy: Progress, challenges, and opportunities: A review. Internat J Biol Macromol.

[4] Niu F., Yu Y., Li Z. (2022). Arginase: an emerging and promising therapeutic target for cancer treatment. Biomed Pharmacother.

[5] Fernandes H., Silva Teixeira C., Fernandes P., Ramos M., Cerqueira N. (2017). Amino acid deprivation using enzymes as a targeted therapy for cancer and viral infections. Expert Opin Ther Pat.

[6] Maggi M., Scotti C. (2019). Enzymes in metabolic anticancer therapy. Therap Enzymes: Funct Clin Impl.

[7] Li M., Qin J., Xiong K., Jiang B., Zhang T. (2022). Review of arginase as a promising biocatalyst: characteristics, preparation, applications and future challenges. Crit Rev Biotechnol.

[8] Chen C.-L., Hsu S.-C., Ann D.K., Yen Y., Kung H.-J. (2021). Arginine signaling and cancer metabolism. Cancers.

[9] Husain I., Bala K., Wani A., Makhdoomi U., Malik F., Sharma A. (2017). Arginase purified from endophytic Pseudomonas aeruginosa IH2: Induce apoptosis through both cell cycle arrest and MMP loss in human leukemic HL-60 cells. Chem Biol Interact.

[10] Riess C., Shokraie F., Classen C.F. (2018). Arginine-depleting enzymes–an increasingly recognized treatment strategy for therapy-refractory malignancies. Cell Physiol Biochem.

[11] Yang J.-S., Wang C.-C., Qiu J.-D., Ren B., You L. (2021). Arginine metabolism: a potential target in pancreatic cancer therapy. Chin Med J (Engl).

[12] Hassabo A.A., Selim M.H., Saad M.M., Abdelraof M. (2022). Optimization of l-methioninase and l-arginase production by newly isolated marine yeast using response surface methodology. Biocatal Agric Biotechnol.

[13] Qader M.M., Hamed A.A., Soldatou S. (2021). Antimicrobial and antibiofilm activities of the fungal metabolites isolated from the marine endophytes Epicoccum nigrum M13 and Alternaria alternata 13A. Mar Drugs.

[14] Sabu A. Sources, properties and applications of microbial therapeutic enzymes. 2003.

[15] Mahjoubi M., Aliyu H., Cappello S. (2019). The genome of Alcaligenes aquatilis strain BU33N: Insights into hydrocarbon degradation capacity. PLoS One.

[16] Javia B., Gadhvi M., Vyas S. (2023). Bioprospecting of a thermostable L-methioninase from Alcaligenes aquatilis BJ-1 in agro-industrial waste. Microbiol Res.

[17] Yadav H., Singh S., Sinha R. (2024). Industrial microbiology and biotechnology: a new horizon of the microbial world.

[18] Osman M.E., Elnasr A.A.A., Mohamed E.T., Faraag A.H. (2024). Enhancement of Streptomyces thinghirensis WAE1 for production of bioactive metabolites under different optimization strategies. Microb Pathog.

[19] Khaswal A., Mishra S.K., Chaturvedi N. (2024). Optimization of process parameters using response surface methodology (RSM) for laccase enzyme production using Aspergillus nidulans in solid state fermentation utilizing agro-industrial waste. J Integ Sci Technol.

[20] Lamidi S., Olaleye N., Bankole Y., Obalola A., Aribike E., Adigun I. (2022). In product design, development, and process optimization.

[21] Unissa R., Sudhakar M., Reddy A.S.K. (2015). Selective isolation and molecular identification of L-arginase producing bacteria from marine sediments. World J Pharm Pharm Sci.

[22] Nadaf P., Kulkarni A.G., Vedamurthy A. (2019). Isolation, screening and characterization of L-arginase producing soil bacteria. Int J Pharm Sci Res.

[23] Kim M., Morrison M., Yu Z. (2011). Evaluation of different partial 16S rRNA gene sequence regions for phylogenetic analysis of microbiomes. J Microbiol Methods.

[24] Farris J.S., Albert V.A., Källersjö M., Lipscomb D., Kluge A.G. (1996). Parsimony jackknifing outperforms neighbor-joining. Cladistics.

[25] Zhang T., Guo Y., Zhang H., Mu W., Miao M., Jiang B. (2013). Arginase from bacillus thuringiensis SK 20.001: purification, characteristics, and implications for L-ornithine biosynthesis. Process Biochem.

[26] Ahn K.-Y., Lee B., Han K.-Y., Song J.-A., Lee D.S., Lee J. (2014). Synthesis of mycoplasma arginine deiminase in E. coli using stress-responsive proteins. Enzyme Microb Technol.

[27] Assega B.G., Getahun K.A., Milkessa T. (2025). Production of extracellular L-arginase by Alcaligenes aquatilis BC2 isolated from soda lakes (Lake Chitu) of Ethiopia. J Ind Microbiol Biotechnol.

[28] Challa S., Neelapu N.R.R. (2019). Essentials of bioinformatics, volume III: In silico life sciences: agriculture.

[29] Bhunia B., Basak B., Dey A. (2012). A review on production of serine alkaline protease by bacillus spp. J Biochem Technol.

[30] Dutta D., Kumar P., Singh A., Khade S. (2024). Bioprocess strategies for enhanced performance in single‐use bioreactors for biomolecule synthesis: a biokinetic approach. Food Bioeng.

[31] Gautam H., Kumari N., Bansal S. (2022). Enhancing the production of therapeutic enzyme arginase from Lactobacillus acidophilus using response surface methodology. Braz Arch Biol Technol.

[32] Agbaje A., Oyeyiola G.P., Sule I.O. (2022). Extraction and quantification of l-arginase produced by Alcaligenes faecalis. J Microbiol Biotechnol Food Sci.

[33] Ibrahim H., Agbaje A., Afolabi H., Usman H. (2018). Effects of temperature, pH, and agitation rate on the production of microbial L-arginase. J Pharm Biol Sci..

[34] Nadaf P., Vedamurthy A.B. (2020). Optimization of l-arginase production by Pseudomonas sp. Strain PV1 under submerged fermentation. Int J Scient Technol Res..

[35] Selim M.S., Mounier M.M., Abdelhamid S.A., Hamed A.A., Abo Elsoud M.M., Mohamed S.S. (2024). Characterization, modeling, and anticancer activity of L. arginase production from marine bacillus licheniformis OF2. BMC Biotech.

[36] Unnisa R., Rekha K., Aamani D., Konakanchi S., Kumar S. (2018). Production of L-arginase under SSF and its optimization. Mintage J Pharm Med Sci.

[37] El-Wahsh HHH, Abo-Dahab NF, El-Shenawy FS, Amara AAAF, El-Baky NA. Isolation of an Arginase-Producing Aspergillus niger AUMC16187 from Sandy Soil at Al-Gharbia Governorate, Egypt and Characterization of the Produced Enzyme. Preprints: Preprints; 2025. DOI:10.20944/preprints202503.0811.v1.

[38] Montgomery D.C., Peck E.A., Vining G.G. (2021).

[39] Chatterjee S., Hadi A.S. (2015).

[40] Anderson M.J., Whitcomb P.J. (2016).

[41] Nathans L.L., Oswald F.L., Nimon K. (2012). Interpreting multiple linear regression: a guidebook of variable importance. Pract Assess Res Eval.

[42] Rajasimman M., Murugaiyan K. (2010). Optimization of process variables for the biosorption of chromium using Hypnea valentiae. Nova Biotechnologica..

[43] Mokhtar N.A.I.M., Ashari S.E., Zawawi R.M. (2023). Optimization of a lipase/reduced graphene oxide/metal–organic framework electrode using a central composite design-response surface methodology approach. RSC Adv.

[44] Bezerra M.A., Santelli R.E., Oliveira E.P., Villar L.S., Escaleira L.A. (2008). Response surface methodology (RSM) as a tool for optimization in analytical chemistry. Talanta.

[45] Chiappini F.A., Azcarate S.M., Teglia C.M., Goicoechea H.C. (2023). Introduction to Quality by Design in Pharmaceutical Manufacturing and Analytical Development: Springer.

[46] Grzelak M., Borucka A., Guzanek P. (2021). International conference innovation in engineering.

[47] Mohammadi A., Nemati S., Mosaferi M., Abdollahnejhad A., Almasian M., Sheikhmohammadi A. (2017). Predicting the capability of carboxymethyl cellulose-stabilized iron nanoparticles for the remediation of arsenite from water using the response surface methodology (RSM) model: modeling and optimization. J Contam Hydrol.

[48] Laghari R.A., Li J., Xie Z., Wang S.-q. (2018). Modeling and optimization of tool wear and surface roughness in turning of Al/SiCp using response surface methodology. 3D Res.

[49] Sharma P., Sharma A.K. (2025). Application of response surface methodology for optimization of fuel injection parameters of a dual fuel engine fuelled with producer gas-biodiesel blends. Energy Sources Part A.

[50] Sahu R., Meghavarnam A.K., Janakiraman S. (2020). Response surface methodology: an effective optimization strategy for enhanced production of nitrile hydratase (NHase) by Rhodococcus rhodochrous (RS-6). Heliyon..

[51] Sanchez S., Demain A.L. (2008). Metabolic regulation and overproduction of primary metabolites. J Microbial Biotechnol.

[52] Montgomery D.C. (2017).

[53] Bremer E., Krämer R. (2019). Responses of microorganisms to osmotic stress. Annu Rev Microbiol.

[54] Abdelraof M., Elsoud M.M.A., Selim M.H., Hassabo A.A. (2020). l-arginine amidinohydrolase by a new Streptomyces isolate: screening and statistical optimized production using response surface methodology. Biocatal Agric Biotechnol.

[55] Momin B., Chakraborty S., Annapure U. (2018). Investigation of the cell disruption methods for maximizing the extraction of arginase from mutant bacillus licheniformis (M09) using statistical approach. Korean J Chem Eng.

